# In vivo antibacterial activity of chitosan nanoparticles against Clostridium perfringens-induced necrotic enteritis in broilers

**DOI:** 10.1186/s13620-026-00342-6

**Published:** 2026-04-13

**Authors:** Reham Elnagar, Akram A. H. Al-Khalidi, Rasha Elkenany, Heba M. M. Abdel-Aziz, Amal Awad, Gamal Younis

**Affiliations:** 1 Department of Bacteriology, Immunology, and Mycology, Faculty of Veterinary Medicine, Mansoura University, Mansoura, 35516 Egypt; 2 Department of Pathology, College of Veterinary Medicine, University of Diyala, Baqubah, Iraq; 3 Botany Department, Faculty of Science Mansoura University, Mansoura, 35516 Egypt

**Keywords:** Chitosan nanoparticles, Necrotic enteritis, *Clostridium perfringens*, Broilers, Antibacterial activity, Histopathology

## Abstract

**Background:**

Necrotic enteritis (NE) is a major disease affecting broiler productivity, primarily caused by Clostridium perfringens. The extensive use of antibiotics to control NE has led to concerns about antimicrobial resistance and food safety, highlighting the need for safe and effective alternatives. Chitosan nanoparticles (CsNPs) have emerged as promising candidates due to their antibacterial, biodegradable, and nontoxic properties. This study aimed to evaluate the therapeutic potential of CsNPs against C. perfringens infection in broilers by assessing clinical signs, mortality, growth performance, bacterial load, immune response, and histopathological changes. Sixty 1-day-old Ross 308 chicks were randomly divided into four groups (15 birds each): G1 (negative control), G2 (C. perfringens-infected), G3 (infected + CsNPs), and G4 (CsNPs only). Birds in G2 and G3 were orally inoculated with C. perfringens (4×10⁸ CFU/mL) for two consecutive days, while CsNPs (250 µg/bird) were administered to G3 and G4. During the experiment, the birds were assessed for growth performance, intestinal microbial loads, immune organ indexes, and histopathological lesions.

**Results:**

Over the 35-day trial, CsNPs treatment significantly improved body weight (BW), body weight gain (BWG), and feed conversion ratio (FCR) in G3 and G4 compared to G2 (P < 0.05). CsNPs reduced clinical signs and mortality and markedly decreased intestinal colonization by C. perfringens. Immune organ indices (spleen, bursa, and thymus) were significantly enhanced in CsNP-treated birds. Histopathological examination revealed that CsNPs mitigated lesions in the intestine, liver, and lymphoid organs.

**Conclusion:**

Chitosan nanoparticles effectively reduced the severity of necrotic enteritis in broilers, improving growth performance, intestinal health, and immune response. These findings indicate that CsNPs could serve as a safe, natural, and sustainable alternative to antibiotics for controlling C. perfringens infections in poultry.

## Introduction

Bacterial infections represent a major health challenge in poultry production, leading to significant economic losses worldwide due to increased mortality, reduced growth performance, and decreased productivity. Infectionse infections, necrotic enteritis (NE) is considered one of the most important enteric diseases affecting poultry. The disease is primarily caused by *Clostridium perfringens*, a Gram-positive, anaerobic, spore-forming bacterium that proliferates in the intestinal tract under favorable conditions and induces severe intestinal damage. According to the four essential toxins produced alpha (α), beta (β), epsilon (ε), and iota (ι), *C. perfringens* strains are classified into five toxinotypes (A to E) [1]. Among these, type A is the most commonly isolated strain in NE outbreaks and is characterized by the secretion of alpha toxin as its principal virulence factor. Type C strains may also be involved, although less common [2]. Notably, all toxinotypes produce alpha toxin, which is regarded as a key virulence factor due to its hemolytic, phospholipase C, and sphingomyelinase properties [3]. The primary mechanism of action of alpha toxin is the hydrolysis of phospholipids in host cell membranes, which causes cell lysis and membrane disarray [4].

Broilers usually develop NE between the ages of two and six weeks, which can result in rapid death without any warning symptoms. The disease is estimated to contribute to 10–40% of annual mortality in poultry populations worldwide, resulting in economic losses to the global poultry industry estimated at USD 2–6 billion annually [5]. The severity of NE is influenced by multiple factors. Together with *C. perfringens’s* virulence factors, co-infections as coccidiosis, stress, immunosuppressive diseases (like infectious bursal disease), and changes in the feed or the additives of feed can significantly alter disease outcomes [6]. NE has re-emerged as a major clinical and subclinical threat in broiler flocks, particularly due to the restriction of using antibiotics in-feed and the adoption of modern high-density housing systems [7‏].

High death rates are a hallmark of the clinical form of NE, which progresses quickly. Common clinical symptoms include apathy, ruffled feathers, reduced feed intake, yellowish foamy diarrhea, and dehydration [8]. Mortality rates during outbreaks of clinical necrotic enteritis may reach up to 50%, with outbreaks typically lasting approximately 5–10 days. The disease often progresses peracutely, and affected birds may die within 1–2 h after the onset of clinical signs, while flock outbreaks generally persist for several days [9].

The subclinical form of necrotic enteritis is generally not associated with increased mortality; however, it negatively affects poultry performance by reducing weight gain and increasing the feed conversion ratio, leading to considerable economic losses in poultry production [9, 10, and 11]. Therefore, effective control and preventive strategies are essential to minimize the economic impact of this disease. Antibiotics have long been the mainstay for the prevention and control of NE. However, there is a growing demand for safe, natural, and effective alternatives due to regulatory restrictions and increasing concerns regarding antimicrobial resistance [12, 13].

Chitosan is a biodegradable and biocompatible natural polysaccharide. Due to its antimicrobial, antioxidant, and chelating properties, it has been widely used in biomedical, pharmaceutical, and veterinary applications as a safe bioactive agent [14]. Activating host defenses, affecting microbes, and forming a layer on the treated surface are the three ways that chitosan demonstrates its protective impact [15].

Nanotechnology can improve the performance and immune health of birds by lowering the microbial burden without leaving drug residues in poultry products and mitigating antibiotic resistance [16]. Unlike conventional antibiotics, many nanomaterials such as chitosan nanoparticles can reduce microbial load without leaving harmful drug residues in poultry products [17]. This is particularly advantageous for food safety, as it lowers the risk of antimicrobial residues entering the human food chain while still effectively controlling pathogens in poultry production. The uses of nano-chitosan, a naturally occurring substance with remarkable physicochemical qualities, have been developed to enhance the microbiota, immunological state, and growth performance of commercial poultry birds [18].

Similar to native chitosan, the antibacterial activity of chitosan nanoparticles (CsNPs) is mainly attributed to their interaction with the bacterial cell wall and membrane. Due to their smaller size and higher zeta potential compared with bulk chitosan, CsNPs exhibit enhanced antibacterial activity and more effectively inhibit bacterial growth [19]. The polycationic nature of CsNPs, resulting from the presence of primary amine groups, enables them to bind to negatively charged bacterial surfaces, disrupt membrane integrity, increase permeability, and ultimately interfere with intracellular components such as DNA, leading to inhibition of replication and bacterial cell death Additionally, CsNPs may inhibit toxin production and modulate gut microbiota by suppressing pathogens while preserving beneficial bacteria [20].

Studies have reported that chitosan nanoparticles (CsNPs) exhibit superior in vitro antibacterial activity compared with chitosan and chitin, effectively inhibiting several clinically important pathogens, including *Escherichia coli*,* Klebsiella pneumoniae*,* Pseudomonas aeruginosa*, and *Staphylococcus aureus* [21]. However, most of the previous studies have mainly focused on the in vitro antibacterial activity of chitosan nanoparticles against different pathogenic microorganisms, while limited information is available regarding their in vivo application, particularly in poultry disease models. In addition, several nanoparticle-based approaches, including metallic nanoparticles such as silver and zinc oxide [22, 23], have been evaluated for controlling *C. perfringens*–induced necrotic enteritis in broiler chickens. Although these nanoparticles demonstrate antibacterial efficacy, concerns remain regarding their potential toxicity and accumulation in edible tissues. Therefore, a significant research gap still exists concerning the evaluation of biodegradable and biocompatible nanoparticles such as chitosan nanoparticles as a safe alternative for controlling *Clostridium perfring*ens infections in poultry. We hypothesized that chitosan nanoparticles could exhibit effective antibacterial activity against *C. perfringens in vivo*. Accordingly, the present study aimed to evaluate the in vivo antibacterial efficacy of chitosan nanoparticles in experimentally infected broiler chickens and to assess their potential as an alternative therapeutic strategy for controlling necrotic enteritis through the assessment of clinical signs, mortality rate, growth performance, bacterial load, immunological responses, and histopathological changes.

## Materials and methods

### Preparation of chitosan nanoparticles

Chitosan nanoparticles (CsNPs) were made using the procedure outlined by [24, 25], which involves methacrylic acid (MAA) polymerization in a solution of chitosan (Cs). Chitosan with a molecular weight (MW) of 1526.46 g mol^**−** 1^ and a deacetylation degree (DD) of 85% was used. Specifically, 0.5% (v/v) methacrylic acid was used to dissolve 0.2 g of chitosan under continuous magnetic stirring for 12 h. The mixture was then gradually mixed with 0.005 g of potassium persulfate while being stirred continuously until a clear solution was obtained. The mixture of reaction was then heated to 70 °C with constant stirring for 1 h to facilitate nanoparticle formation. The pH of the prepared solution was 4.5. To terminate the polymerization process, an ice bath was used to quickly cool the mixture.

### Characterization of chitosan nanoparticles

#### Transmission electron microscope (TEM)

A JEOL 1010 transmission electron microscope (TEM) running at an accelerating voltage of 80 kV was used to examine the shape and size of the produced CsNPs (0.1 mg/mL) (JEOL, EM Unit, Mansoura University). Prior to imaging, the nanoparticle suspension was sonicated using a probe sonicator with 40% amplitude for 2 min to minimize agglomeration and ensure uniform dispersion of particles. After being applied to a carbon-coated copper grid and left to air-dry at room temperature, a single drop of the CsNPs suspension was inspected under a TEM. Using Image ProPlus 4.5 software, the particle sizes were measured straight from the micrographs, following the approach clarified by [26].

#### Zeta potential of chitosan nanoparticles

The zeta potential of CsNPs (in deionized water, 0.1 mg/mL, pH 4.5) was determined using a Zetasizer (Malvern Instruments Ltd., EM Unit, Mansoura University). Prior to measurement, the zeta cell was thoroughly cleaned by sequential rinsing: first with distilled water, followed by ethanol, and finally with water again. The cell was then gently dried using a stream of nitrogen gas to eliminate residual solvents and immediately covered to avoid dust contamination. CsNPs samples were carefully introduced into the zeta cell using a 1 cm³ syringe. Each sample was measured in triplicate to ensure repeatability of the results. The applied voltage during measurements was automatically adjusted by the instrument, as described by [27].

#### Fourier transform infra-red (FT-IR) analysis for chitosan nanoparticles

The Fourier-transform infrared (FT-IR) spectrum of CsNPs was recorded using a NICOLET IS10 FT-IR spectrometer (Mansoura University) over the wavenumber range of 4000–400 cm⁻¹. Prior to analysis, A freeze-drying method was used to lyophilize the nanoparticles after they had been frozen in liquid nitrogen. The resulting dry powder was then mixed with potassium bromide (KBr) to form pellets suitable for FT-IR measurement, following the way described by [24].

#### *Clostridium perfringens* strain

A field strain of *C. perfringens* type A (PV085770) was acquired from Mansoura University’s Faculty of Veterinary Medicine’s Department of Bacteriology, Immunology, and Mycology. After inoculating cooked meat media with the *C. perfringens* type A strain under aseptic circumstances, the mixture was incubated for eighteen hours at 37 °C under anaerobic condition (AnaeroPack-Anaero Kits, Mitsubishi Gas Chemical Inc., Japan).Following their resuspension in phosphate buffered saline (PBS), the bacterial cells were counted using a McFarland tube. The chicks in the CsNPs-treated group and the positive control group were infected orally with 4 × 10^8^ CFU/mL/bird of freshly prepared *C. perfringens* in PBS on two consecutive days at 14 day old in line with the approach outlined by [22].

#### Experimental chicks

This investigation was experimentally designed using sixty-one-day-old Ross308 chicks that were purchased from commercial poultry company, Egypt. Under normal environmental and sanitary settings, the chicks were raised on a litter system with bedding made of fresh wood shavings and food given on an as-needed basis. All of the chicks received the Hitchner B1 + H120 and LaSota vaccines (Intervet Boxmeer Company, Boxmeer, Netherlands) at 5 and 14 days of age, respectively, to prevent Newcastle disease. Predisposing elements must be added in order to cause necrotic enteritis in experimental models of *C. perfringens* infection. To predispose the intestines for successful challenge, the birds were fed a beginning diet devoid of antibiotics that had 20% proteins (w/w) until day 10. From day 11, they were switched to a high-protein diet that consisted of 40% fishmeal protein and 50% wheat.

#### Experimental design

This study was conducted to evaluate the antimicrobial effect of CsNPs. The chicks were randomly divided into four groups (G1 to G4) with five chicks in each group (replicates for each group). The groups (G1-G4) were 1st blank negative control (G1, C), 2nd infected orally with *C. perfringens* (G2, I) (1 ml of 4 × 10^8^ *C. perfringens* at day 14 & 15), 3rd infected and treated with chitosan nanoparticle (G3, I+ CsNPs) (50 µg CsNPs in 1 mL PBS for 5 successive days), 4th treated with chitosan nanoparticle (G4, CsNPs). Beginning at 14 days old, the treatment groups (G3 and G4) received oral gavages of 1 mL of chitosan nanoparticle (50 µg/ml) for 5 days in a row, for a total dose of 250 µg of CsNPs per bird [23]. Clinical signs and mortality were recorded every day. Birds were weighed at 2nd, 3rd, 4th, and 5th week to adjust evaluate body weight gain, feed intake, and feed conversion ratio. Also, intestinal samples and immune organs (Bursa of Fabricius (BF), spleen, and thymus) were gathered on day 21, 28, and 35 for re-isolation of *C. perfringens* and determination of immune organ indices, respectively. Moreover, when the 35-day experiment was over, birds were sacrificed and portions of the internal organs (liver, intestine, spleen, thymus, BF) were dissected for histological examination.

#### Bacterial quantification

To assess *C. perfringens* shedding and colonization, three birds were purposefully chosen at random and humanely killed from each group (one bird per replicate) at 7, 14, and 28 days post-infection. Serial dilutions of intestinal suspensions were spread tryptose sulphite cycloserine agar base supplemented with D-cycloserine (HiMedia, Maharashtra, India) and contained egg yolk emulsion. The plates were kept under anaerobic conditions at 37 °C for twenty-four hours using anaerobic kits and Gas packs anaerobic jars to calculate the viable bacterial colony per gram of sample. The colonies were verified and chosen following [28].

### Immunological assessment

The thymus, spleen and bursa of Fabricius (BF) were used as indicators for the immune system. The immunological organs were collected and weighed in the manner described below to calculate their indices (%):

The immune organ index is calculated as immune organ weight (g) times 100/live body weight (g).

### Histopathological investigation

At the end of the experimental period (day 35), after recording growth performance, three birds per group were randomly selected, weighed, and humanely euthanized. The liver, jejunum, spleen, thymus, and bursa of Fabricius were collected and fixed in 10% neutral buffered formalin for histopathological analysis. Tissue slices were prepared in paraffin and stained with hematoxylin and eosin before microscopic investigations in accordance with instructions outlined by [29].

### Statistical analysis

Software called SPSS Version 23.0 (SPSS, Chicago, IL, USA) was utilized for statistical analysis, and the data was evaluated using one-way analysis of variance. The means were compared using the Tukey’s p test when a significant difference was found. *P* < 0.05 was used to determine the significant outcome. The findings were represented using the means ± standard error. To make chart graphics, the Prism application (Graph-Pad Software, San Diego, CA) was utilized.

## Results

### Chitosan nanoparticle characterization

#### Morphology and size of chitosan nanoparticles suspension at pH 4.0

**Fig. 1:**
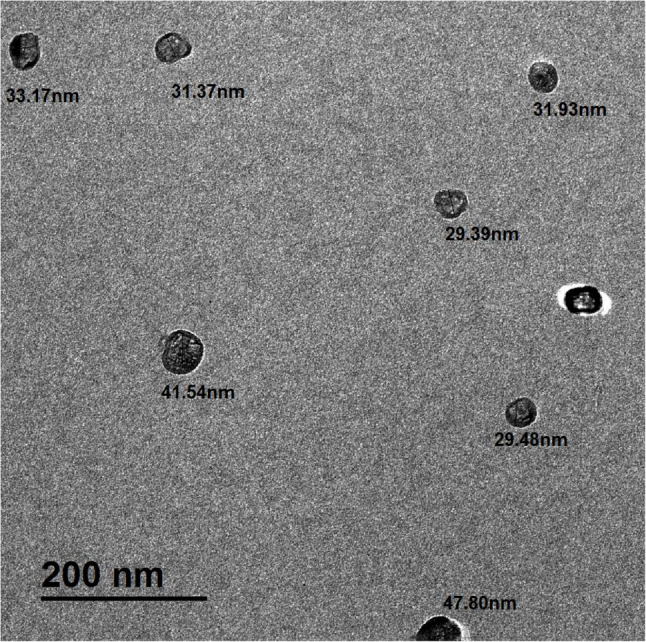
Transmission Electron Micrograph (TEM) of Chitosan Nanoparticles (CsNPs). The nanoparticles were prepared via polymerization with methacrylic acid in deionized water (0.1 mg/mL) following probe sonication (40% amplitude, 2 min) to ensure monodispersion. The morphology reveals distinct, quasi-spherical nanostructures with a relatively uniform size distribution (mean diameter: 32 ± 2)

Transmission electron microscopy of the prepared CsNPs revealed spheres with an average diameter of 32 ± 2 nm (Fig. 1).

### Electrical and chemical properties of chitosan nanoparticles zeta potential

The prepared chitosan nanoparticles’ stability was verified by measuring the zeta potential (ZP ζ) for CsNPs at pH 4.0. The average zeta potential of the produced chitosan nanoparticles was + 30.10 mV.

### Fourier transform infra-red (FT-IR) analysis for CsNPs

**Fig. 2:**
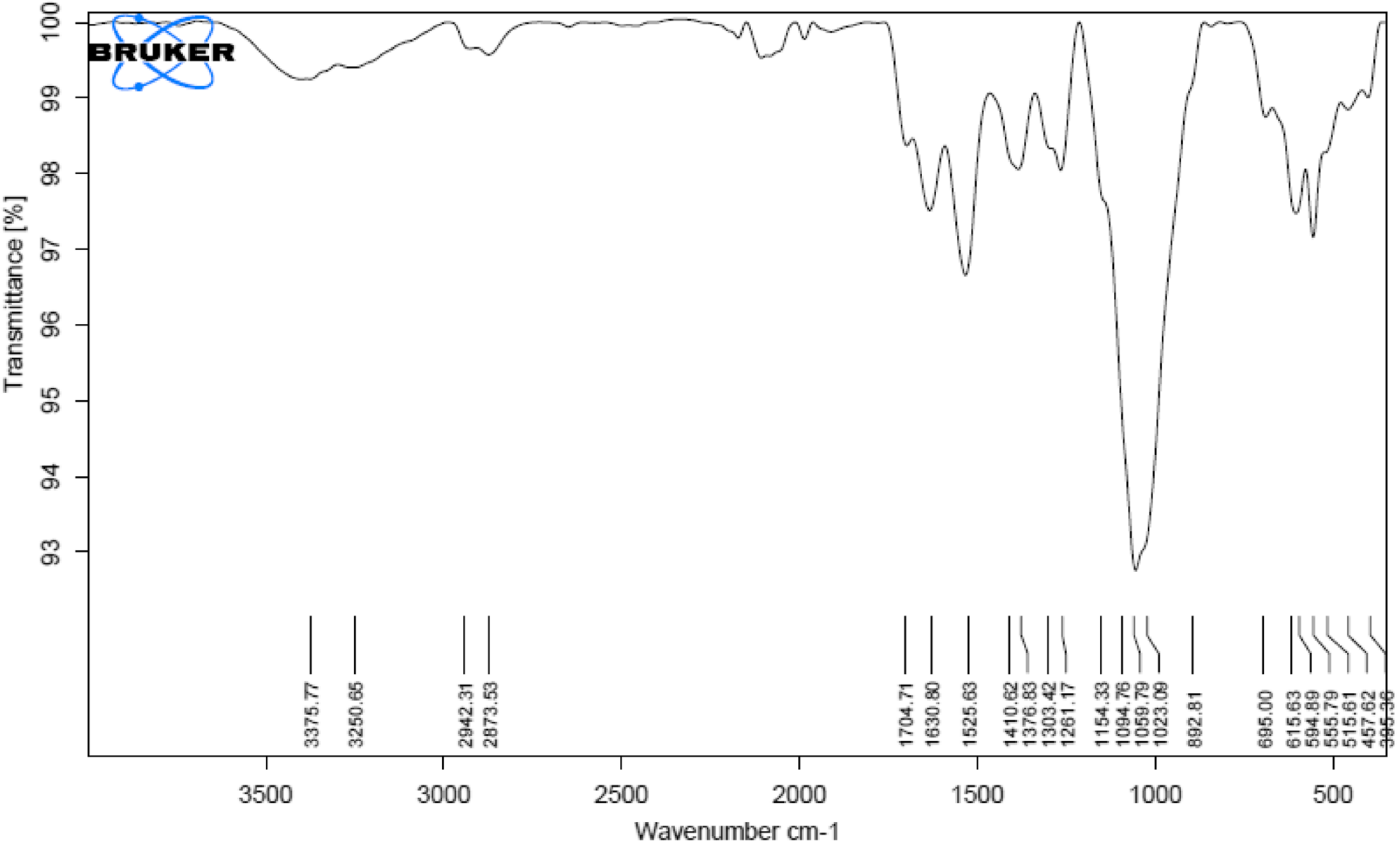
FTIR spectrum for the obtained Chitosan nanoparticles

The presence of functional groups was determined by FT-IR analysis, which examined spectral shifts in position or shape that took place in the distribution of CsNP frequencies. It is evident that the ionic interaction between MAA and Cs linked to the production of nanoparticles is shown by the presence of two additional bands at 1646 and 1546 cm^− 1^ that are attributed to COO − and NH_3_^+^groups, respectively. The existence of poly-MAA in the composition of the nanoparticles is confirmed by the bands at 1741 and 1835 cm^− 1^ (C = O) (Fig. 2).

### Clinical manifestations and the death rate

Clinical symptoms of experimental infection became evident in G2 birds between 21 and 28 days of age. These symptoms included anorexia, ruffled feathers, general depression, and the appearance of dark orange droppings in 40% challenged birds. While the other two groups (G1 and G4) did not exhibit any clinical indicators, 13% of the birds in G3 displayed the observed clinical signs. The clinical signs were less severe in the chicks treated with CsNPs (G3) than in the groups infected with *C. perfringens.* G2 and G3 had respective death rates of 26.6% (4/15) and 6.6% (1/15) at one-week post-infection. Over the course of the experiment, it has been shown that neither the CsNPs treated nor the negative control groups experienced any mortality.

### Growth performance

The growth performance metrics were shown in Table (1). BW, BWG, FI, and FCR were significantly (*P* < 0.05) different in the negative control birds, infected controls, and CsNPs treated groups at weeks 2, 3, 4, and 5. Remarkably, significant (*P* < 0.05) improvements were observed in the body weights of the CsNPs treated group and the CsNPs/Clostridium infected groups as compared to the positive birds. In comparison to G2, the BW and BWG rose linearly with age, with G1 and G4 having the most elevated values over every week of the experiment. From days 14 to 35, the FCR of the birds who received CsNPs (G3 and G4) showed significant improvements (*P* < 0.005). The four experimental groups’ food conversion rates were evaluated. When G3 and G4 birds were given CsNPs, their body gain and food conversion rate were better than those of the control-negative (G1) and control-positive (G2) birds as shown in (Table 1). These results provided credence to chitosan nanoparticles’ potential as a natural growth enhancer and a practical substitute strategy to lessen the detrimental impacts of a *C. perfringens* infection in chicken.

**Table 1:** Effect of chitosan nanoparticle treatment on body weight, body weight gain, feed intake, and feed conversion ratio in challenged chickens

Group	Body weight at age /kg				Cumulative body weight gain/kg	Cumulative Feed intake kg	Cumulative FCR
	2nd week	3rd week	4th week	5th week			
G1	0.33 ± 0.01^b^	0.87 ± 0.01^a^	1.47 ± 0.04^a^	2.20 ± 0.10^a^	1.87 ± 0.10^a^	2.78 ± 0.01^b^	1.49 ± 0.07^b^
G2	0.28 ± 0.01^c^	0.64 ± 0.02^b^	1.06 ± 0.04^b^	1.72 ± 0.09^b^	1.44 ± 0.09^b^	2.89 ± 0.02^a^	2.03 ± 0.15^a^
G3	0.31 ± 0.01^bc^	0.79 ± 0.04^a^	1.43 ± 0.02^a^	2.23 ± 0.15^a^	1.92 ± 0.15^a^	2.82 ± 0.01^b^	1.49 ± 0.12^b^
G4	0.43 ± 0.00^a^	0.87 ± 0.02^a^	1.49 ± 0.03^a^	2.54 ± 0.07^a^	2.11 ± 0.07^a^	2.67 ± 0.03^c^	1.27 ± 0.04^b^
P value	0.000	0.000	0.000	0.004	0.013	0.000	0.005

*Abbreviations*: *FCR* feed conversion ratio (g of feed/g of body weight gain), *SEM* standard error of mean

a,b, c Mean values or percentages with different superscripts in the same column are significantly different (*P* < 0.05). Treatments: G1 chicks were not challenged and received no CsNP treatment; G2 chicks were infected with *C. perfringens*; G3 chicks were infected with *C. perfringens* and received CsNPs treatment; and G4 chicks were uninfected and received CsNPs treatment.

#### C. perfringens intestinal counts

**Fig. 3:**
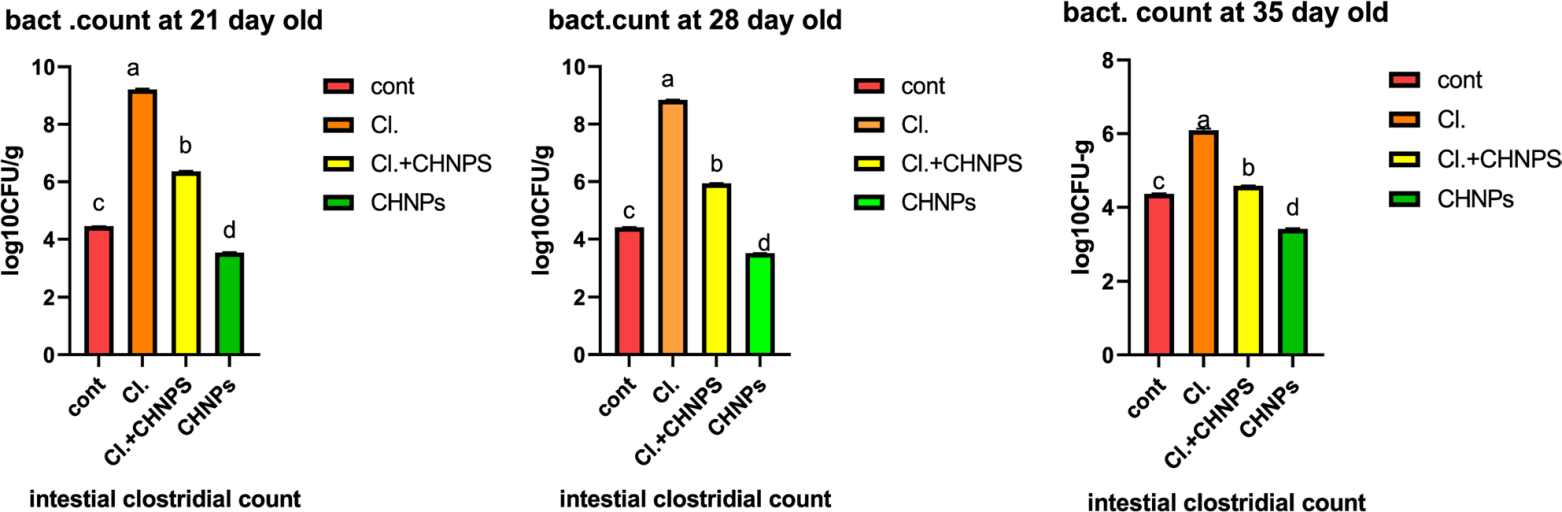
Effect of CsNPs on intestinal *C. perfringens* counts (log10 CFU/g) in broiler chickens. Cont: negative control group(C); Cl: *Clostridium* challenged group (I); Cl+CHNPs: *Clostridium* infected group and treated by chitosan nanoparticles group (I+ CsNPs); CHNPs: non infective and treated by chitosan nanoparticles group (CsNPs). a, b,c, d: Significant differences were indicated by various superscripts (Tukey’s test; *P* < 0.05)

Broiler chicken intestinal counts of *C. perfringens* (log₁₀ CFU/g) were provided in Fig. (3). Throughout all sampling days, the findings demonstrated that the challenged group (I) had a significantly greater bacterial count than all other groups (*p* < 0.05). Conversely, the group treated with chitosan nanoparticles following infection (I + CsNPs) confirmed a significant decrease in bacterial counts in comparison to the infected one (*p* < 0.05). The non-infected group treated with CsNPs on my own exhibited the lowest bacterial counts amongst all groups, with values of 3.54, 3.51, and 3.41 at the respective time factors. These values have been even lower than those detected in the negative control group. These effects imply that experimental infection with *C. perfringens* type A led to an extensive growth in intestinal bacterial counts in broiler chickens, confirming the pathogenicity of the bacterial strain used. The treatment of chitosan nanoparticles (CsNPS) extensively decreased the intestinal bacterial load in both infected and non-infected birds, demonstrating effective antibacterial activity.

### Immunological Assessment

The bursa of Fabricius, spleen and thymus’s relative weights have been significantly affected by *C. perfringens* infection and remedy with CsNPs across all time points (days 21, 28, and 35) (Table 2). The spleen, bursa, and thymus indices showed significant variations between treatment groups (*P* < 0.05). Throughout the investigation, the chicks, who were given CsNPs alone (G4) had the highest immune organ indices (*p* < 0.05), suggesting that chitosan nanoparticles had a potent immunostimulatory impact. Conversely, the *C. perfringens* infected group displayed the lowest values, indicating that *C. perfringens* infection suppresses the development of immunological organs. In contrast to the infected group, the infected and treated group (G3, I+CsNPs) showed a significant improvement in immune organ weights, indicating a protective effect of CsNPs against the infection, even if the values were still just slightly below the CsNPs. The negative control group exhibited moderate values that were better than the infected group but lower than CsNPs. Therefore, CsNPs showed both immunostimulatory and protective effect in broilers counteracting the suppressive impact of *C. perfringens.*

**Table 2:** Impacts of chitosan nanoparticles treatment on immune organ indexes of broilerchickens at different ages

Groups	Spleen			Bursa of Fabricius			Thymus		
	D21	D28	D35	D21	D28	D35	D21	D28	D35
G1	0.099 ± 0.002^b^	0.101 ± .0006^b^	0.111 ± .002^b^	0.323 ± 0.008^a^	0.23 ± 0.002^a^	0.155 ± 0.002^b^	0.463 ± 0.005^a^	0.402 ± 0.006^b^	0.33 ± 0.006^b^
G2	0.062 ± 0.0015^d^	0.07 ± 0.002^d^	0.082 ± .002^c^	0.173 ± 0.004^c^	0.153 ± 0.001^c^	0.115 ± 0.003^c^	0.352 ± 0.004^c^	0.291 ± 0.0007^d^	0.241 ± 0.003^c^
G3	0.089 ± 0.001^c^	0.092 ± 0.0004^c^	0.109 ± 0.0008^b^	0.255 ± 0.005^b^	0.202 ± 0.005^b^	0.151 ± 0.001^b^	0.419 ± 0.004^b^	0.361 ± 0.003^c^	0.324 ± 0.004^b^
G4	0.113 ± 0.001^a^	0.122 ± 0.001^a^	0.130 ± 0.0005^a^	0.347 ± 0.012^a^	0.241 ± 0.007^a^	0.175 ± 0.006^a^	0.48 ± 0.002^a^	0.451± .003^a^	0.360 ± 0.003^a^

Each group consists of three replicates (15 birds). *P* < 0.05 indicates significant differences between means (Mean ± SE) with different lowercase letters in the same column. SE: standard error.

a, b, and c  Significant differences (*P* < 0.05) exist between mean values in the same column that have distinct superscripts.G1, chick neither infected nor treated with CsNPs (C); G2, C. perfringens-challenged group  (I); G3, C. perfringens -challenged, followed by CsNPs treatment group (I+ CsNPs); G4, uninfected chicks treated with CsNPs.

### Histopathology

**Fig. 4:**
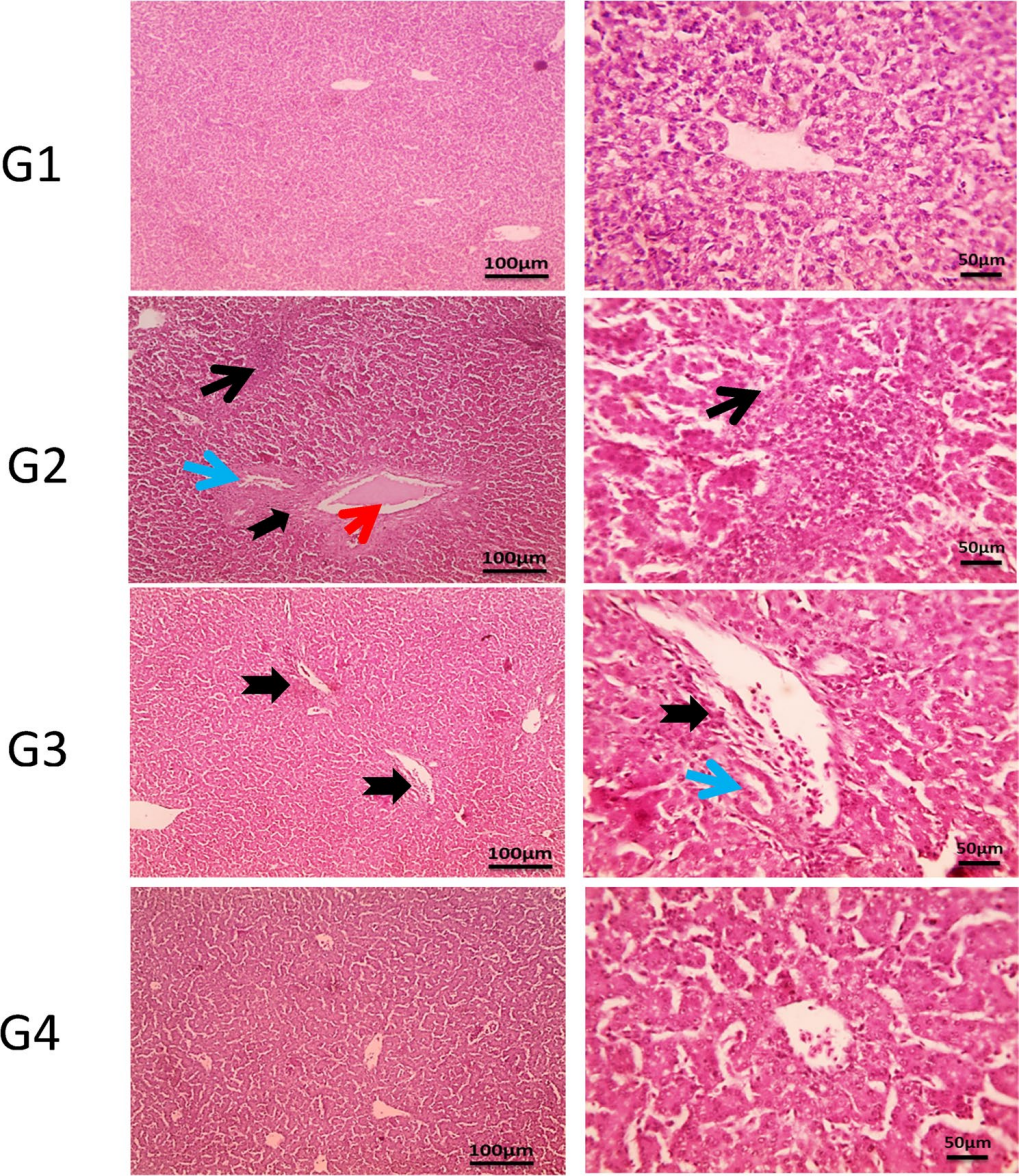
Histopathology sections of liver. G1 (control) &G4 (CsNPs): normal histological structure consisted of hepatic cords, sinusoids, central veins and portal areas. G2 (C. perfringens-infected): showing multifocal aggregation of heterophils replacing hepatic parenchyma (thin black arrow), besides, extended portal areas with fibrosis & inflammation (thick black arrow), congestion of portal vein (red arrow), proliferated and dilated bile ductile (blue arrow). G3 (Infected+ CsNPs): mild portal fibrosis & inflammation (thick black arrow). H&E, magnifications X: 100 bar 100 and X: 400 bar 50

**Fig. 5:**
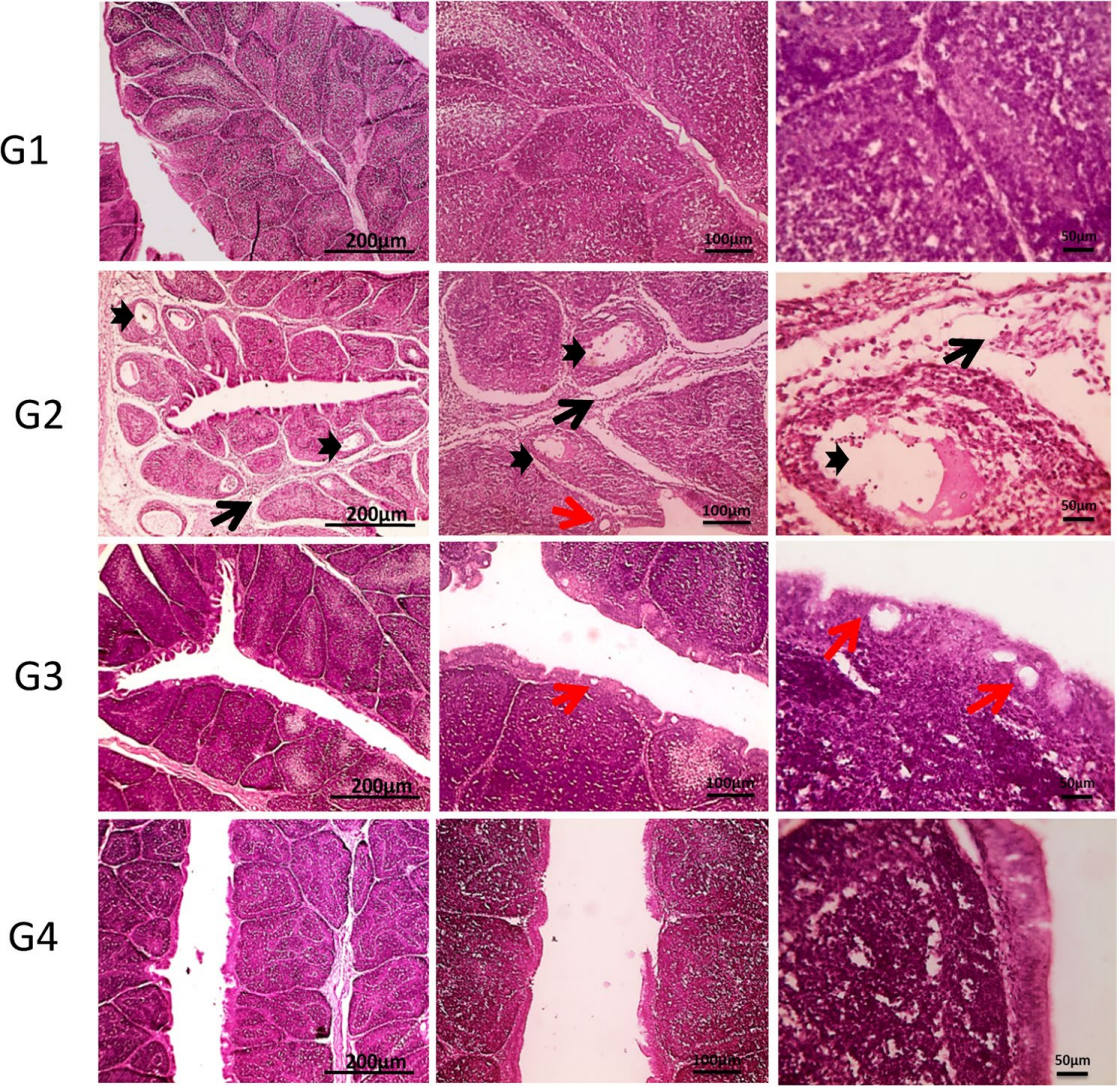
Histopathology sections of Bursa of Fabricius. G1 (control) &G4 (CsNPs): normal histological structure of folia each lined by columnar epithelium and contain many large lymphoid follicles consist of cortex and medulla. G2 (C. perfringens-infected): cavitation of follicular medulla due to necrosis of lymphocytes that is replaced by eosinophilic homogenous material (thick black arrow), interstitial edema and leukocytic cells infiltration mainly heterophils (thin black arrow), vacuoles formation in lining epithelium (red arrow). G3 (Infected+ CsNPs): normal large lymphoid follicles with few vacuoles’ formation in lining epithelium (red arrow). H&E, magnifications X: 40 bar 200, X: 100 bar 100 and X: 400 bar 50

**Fig. 6:**
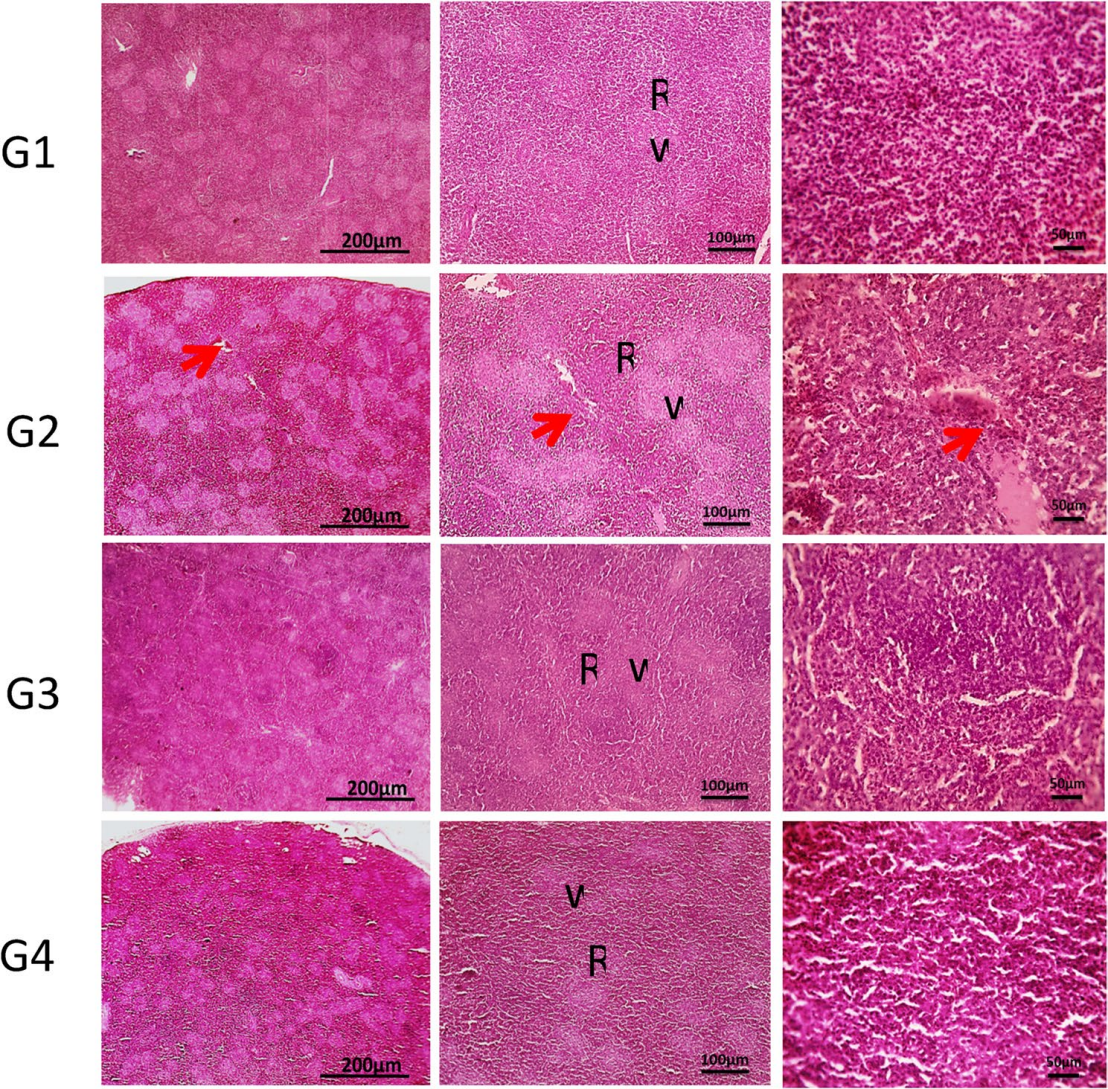
Histopathology sections of spleen. G1 (control) &G4 (CsNPs): normal histological structure of white (w) and red pulp (R). G2 (C. perfringens-infected): congestion in red pulp (red arrow). G3 (Infected+ CsNPs): normalized histological picture of white and red pulp. HE, magnifications X: 40 bar 200, X: 100 bar 100 and X: 400 bar 50

**Fig. 7:**
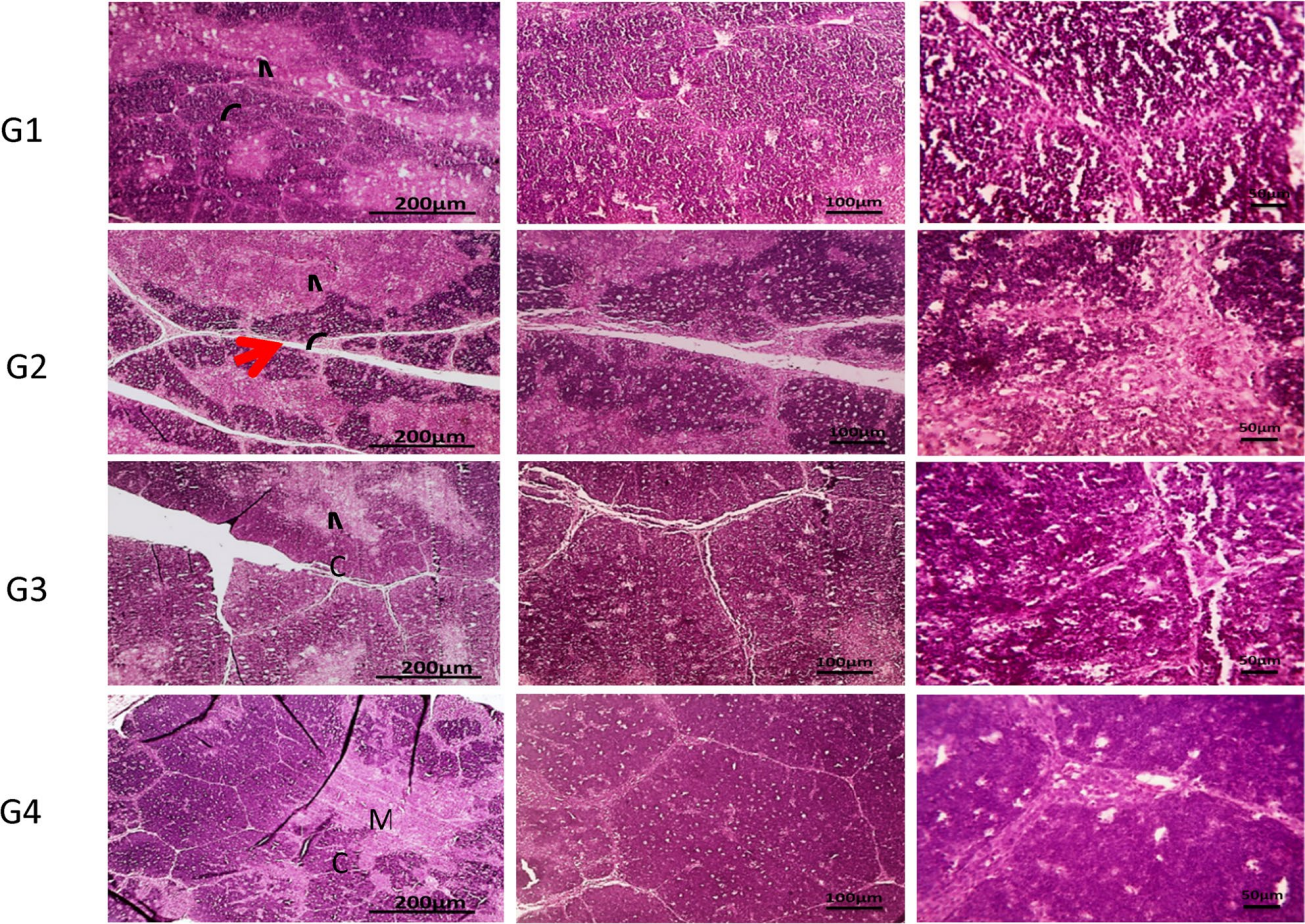
Histopathology sections of thymus. G1 (control) &G4 (CsNPs): normal histological structure of cortex (C) and medulla (M). G2 (C. perfringens-infected): markedly decreased thickness of cortex. G3 (Infected+ CsNPs): increased thickness of cortex. HE, magnifications X: 40 bar 200, X: 100 bar 100 and X: 400 bar 50

**Fig. 8:**
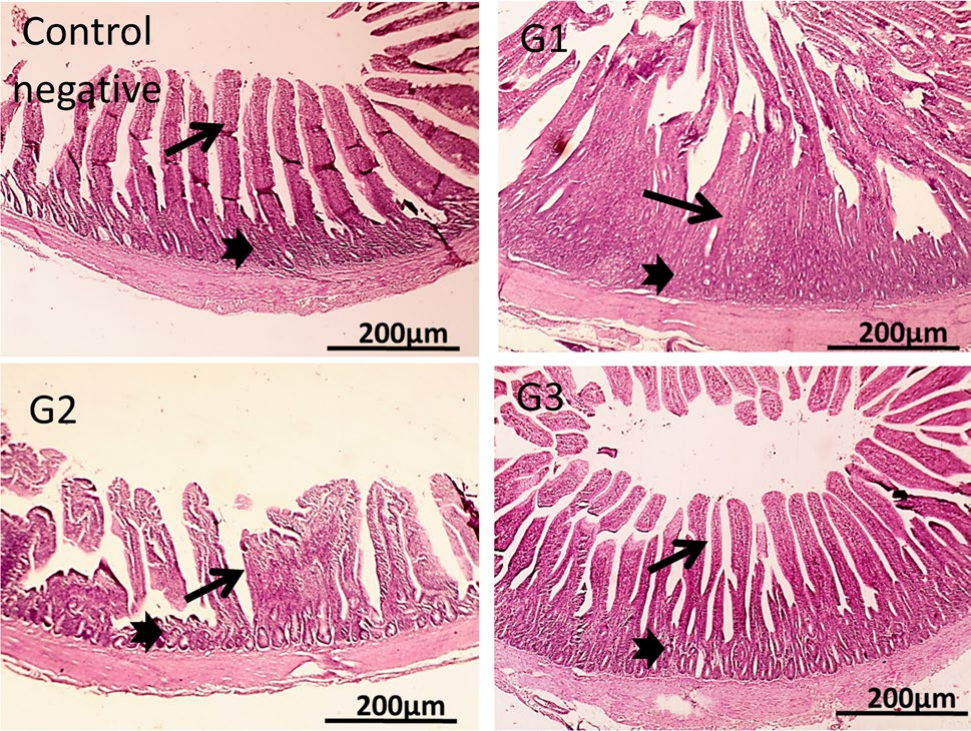
Histopathology sections of Jejunum. G1 (control) &G4 (CsNPs): normal histological structure of villi (thin arrows) and crypts (thick arrows). G2 (*C. perfringens*-infected): markedly fused villi (thin arrows) and proliferated crypts (thick arrows). G3 (Infected+ CsNPs): few fused villi (thin arrows) and normal crypts (thick arrows). HE, magnification X: 40 bar 200

Figure 4 demonstrates the hepatic cords, sinusoids, central veins and portal areas of normal liver of the control group (G1). In G2, multifocal aggregation of heterophils replacing hepatic parenchyma besides, extended portal areas with fibrosis & inflammation, congestion of portal vein, proliferated and dilated bile ductile occurred. In G3, mild portal fibrosis & inflammation was shown. Bursa of Fabricius in Fig. 5, cavitation of follicular medulla due to necrosis of lymphocytes, interstitial edema and leukocytic cells infiltration mainly heterophils, vacuoles formation in lining epithelium was shown in G2. In **G3**, normal large lymphoid follicles with few vacuoles formation in lining epithelium were observed. Furthermore, Fig. 6 reveals congestion in red pulp of spleen in G2 in comparison to G3 that showed normal white and red pulp. Moreover, Fig. 7 demonstrates markedly increase in thickness of thymus cortex in G3 in comparison **to** G2. The G2 jejunum showed markedly fused villi and proliferated crypts, while G3 revealed few fused villi and normal crypts **(**Fig. 8**).** Additionally, the bursa of Fabricius, liver, spleen, thymus, and jejunum of the G4 (CsNPs-treated group) showed normal histological structure.

## Discussion

Multidrug-resistant bacteria have emerged largely due to the extensive use of antibiotics as feed additives, making the search for safe and effective alternatives a global priority. Several nanoparticle formulations have previously been reported to exhibit potent and safe immunomodulatory activities in vitro [30]. In Egypt, numerous studies have investigated the in vitro antibacterial activity of chitosan nanoparticles (CsNPs) against diverse pathogens [31, 32]. To the best of our knowledge, the current study represents the first clinical trial conducted in Egypt to investigate the potential mechanisms of action of CsNPs against necrotic enteritis (NE) in broilers caused by *C. perfringens*. In the present study, oral administration of CsNPs to infected broilers improved feed conversion ratio, exerted protective impacts on immune organs, enhanced tissue histopathology, and significantly decreased intestinal shedding of *C. perfringens*. Collectively, these improvements contributed to effective protection against NE in clinically challenged broilers. The enhanced antimicrobial activity of CsNPs may be attributed to their nanoscale size (< 100 nm), which enables strong antimicrobial, immunomodulatory, and antioxidant properties while also improving gut microbial balance and growth performance [33]. Furthermore, CsNPs exhibit greater biological action than conventional chitosan due to the advantages of nanonization, including enhanced absorbability and improved chemical solubility [34].

The morphology and size of the chitosan nanoparticle suspension at pH 4.0 were consistent with the mechanism previously described by [25, 35]. During polymerization of MAA, the carboxyl groups (-COOH) of polymethacrylic acid (PMAA) interact with the amino groups (-NH3⁺) of chitosan, resulting in intra- and intermolecular bonding that leads to nanoparticle formation. Additionally, the colloidal suspension of CS-PMAA carries a positive charge, and the presence of positive ions can influence the stability of the suspension [26]. The zeta potential (ζ-potential), which reflects the surface charge of nanoparticles in solution, is closely associated with nanosuspension stability. Low potential values may lead to flocculation or coagulation due to van der Waals inter-particle attractions, whereas higher positive or negative values enhance electrical stability of the suspension [36].

Fourier transform infrared spectra were obtained in the range of 400–4000 cm⁻¹. Depending on the synthesis method, CsNPs may contain different functional groups such as C = O, OH, and COOH, which can be modified by oxidation or eliminated through heat treatment. The appearance of two new bands at 1646 and 1546 cm⁻¹, corresponding to COO⁻ and NH3⁺ groups, indicates ionic interactions between MAA and chitosan during nanoparticle formation. In addition, bands observed at 1741 and 1835 cm⁻¹ (C = O) confirm the presence of poly-MAA within the nanoparticle structure [37, 38].

In the present investigation, infected birds treated with CsNPs exhibited reduced severity of clinical signs and a markedly lower mortality rate (6.6%) compared with infected untreated birds. Similarly [39], reported that experimentally infected birds with *C. perfringens* developed severe clinical symptoms, including ruffled feathers, orange foamy droppings, and general depression, with mortality reaching 7.41 %. In addition, birds treated with CsNPs, whether infected or non-infected, showed significant improvements in BW, BWG, and FCR compared with untreated birds. Previous studies have also demonstrated that *C. perfringens* infection negatively affects growth performance in broiler chickens [8, 40]. Reviews evaluating the effects of CsNPs on broiler health and productivity have similarly reported improvements in BWG, final body weight (FBW), and FCR following CsNP supplementation [41, 42].

CsNPs promote growth and enhance efficiency through various mechanisms, including improved immune function, enhanced intestinal integrity, increased antioxidant capacity, and improved nutrient digestibility [43]. In addition, their antimicrobial effect against intestinal pathogens contributes to better nutrient absorption, thereby promoting growth and development [44]. These beneficial effects may clarify the improved body weight and feed conversion ratio observed in birds treated with CsNPs in the present study.

The infected-treated group also showed a significant reduction in bacterial counts compared with the infected untreated group (*p* < 0.05). Interestingly, birds receiving CsNPs alone exhibited the lowest bacterial counts among all experimental groups, even lower than those observed in the uninfected negative control group. Previous studies have demonstrated that chitosan nanoparticles significantly reduce several pathogenic bacteria, including *S. Kentucky*, *P. aeruginosa*, *S. aureus*, and coliform bacteria [31, 45]. Moreover, chitosan supplementation has been shown to increase the population of beneficial bacteria, particularly *Lactobacillaceae*, thereby improving gut microbial ecology in broiler chickens. These beneficial microbes produce lactic and acetic acids, which lower intestinal pH and inhibit the growth of pathogenic bacteria [46]. The strong antibacterial efficacy of CsNPs is largely attributed to their small particle size and large surface area, which enhance their biological reactivity [19]. Collectively, these findings highlight the potential of CsNPs as a safe and effective antibacterial agent for controlling bacterial infections such as *C. perfringens* while maintaining intestinal health in poultry.

The spleen, thymus, and bursa of Fabricius are key components of the immune system in broilers and play essential roles in immune responses and physiological homeostasis [47]. In the present study, *C. perfringens* infection significantly reduced the indices of these immune organs, with the thymus index showing the most pronounced decrease (*p* < 0.05) [48]. Similar alterations in immune organs following *C. perfringens* infection have been previously reported, including changes in morphology and weight of the bursa of Fabricius, spleen, and thymus [49]. This immunosuppressive effect may be attributed to the action of alpha toxin, a potent cytolytic factor that disrupts host cell membranes and induces tissue damage [50].

Importantly, CsNP supplementation demonstrated protective and stimulatory effects on immune organs, restoring the thymus, spleen, and bursa indices that were reduced following *C. perfringens* challenge. These results suggest that CsNPs possess both protective and immunostimulatory properties that counteract the immunosuppressive effects of *C. perfringens*. This activity may be associated with the reactive amino and hydroxyl groups of CsNPs, which stimulate antibody and lysozyme production and activate macrophages [51]. Supporting evidence has shown that supplementation with 100 mg/kg CsNPs-Cu significantly increased spleen, thymus, and bursa indices (*p* < 0.05) [52], while administration of 50 mg/L CsNPs improved the bursa index, indicating enhanced immune responsiveness [53].

Histopathological examination in the current study further confirmed the protective effects of CsNPs treatment against *C. perfringens* infection in broiler organs, including the liver, intestine, and immune organs (bursa of Fabricius and thymus). Infection with *C. perfringens* caused marked damage to intestinal villi and hepatic tissues, consistent with previous findings [22, 48]. In contrast, birds treated with CsNPs following infection showed clear improvements in intestinal villi and liver tissue integrity, with preserved structural organization and reduced inflammatory responses, reflecting the antimicrobial activity of CsNPs. In addition, pathological lesions in the spleen were alleviated, and normal architecture of red and white pulp was restored. The bursal follicles appeared large and well organized with minimal epithelial vacuolation, while the thymus exhibited increased cortical thickness. These observations indicate restoration of normal histological architecture in CsNPs-treated birds and reflect an improved health status. Similarly [54], reported that chitosan enhances small intestinal architecture by increasing villus height, villus width, and the villus height-to-crypt depth ratio, thereby improving nutrient absorption in broilers. Another mechanism by which chitosan supports intestinal morphology and function is through reducing oxidative stress and excessive inflammatory responses in stressed chickens [55]. Collectively, these findings support the immunomodulatory and protective properties of CsNPs; however, further studies are required to establish standardized guidelines for CsNP supplementation in broiler diets. Despite the promising findings of the present study, several limitations should be acknowledged. The experiment was conducted under controlled experimental conditions and involved the evaluation of a single dosage of chitosan nanoparticles. Therefore, the optimal dosage and long-term safety of CsNPs were not investigated. In addition, the precise molecular mechanisms underlying the antibacterial and immunomodulatory effects of CsNPs were not fully explored. Consequently, further studies are required to evaluate different dosage levels, investigate long-term safety, and assess the practical application of CsNPs under large-scale commercial poultry production systems.

## Conclusion

This study provides initial evidence of the in vivo therapeutic potential of chitosan nanoparticles (CsNPs) against *Clostridium perfringens* infections in broilers in Egypt. Treatment with CsNPs was associated with a reduction in the severity of clinical signs, improved growth performance and immune responses, and decreased bacterial colonization and histopathological lesions compared with untreated or control groups. Birds receiving CsNPs also showed improvements in immune organ indices, suggesting a possible protective effect against *C. perfringens* infection. While these results indicate that CsNPs may help mitigate necrotic enteritis and support immune function in broilers, further studies with larger sample sizes, different doses, and field conditions are needed to confirm their efficacy and safety. Overall, CsNPs show promise as a potential alternative or complementary strategy to traditional antibiotics in poultry production.

## Data Availability

No datasets were generated or analysed during the current study.
